# Sex-specific patterns of valvulo-aortopathy in bicuspid aortic valve according to the international consensus framework: insights from the RE-BAV registry

**DOI:** 10.1093/ehjci/jeag233

**Published:** 2026-08-31

**Authors:** Edyta Płońska-Gościniak, Wojciech Kosmala, Jerzy Pacholewicz, Celina Wojciechowska, Ilona Kowalik, Bożena Sobkowicz, Tomasz Hirnle, Andrzej Tomaszewski, Beata Zaborska, Maria Binkiewicz-Orluk, Andrzej Szyszka, Tomasz Hryniewiecki, Julia Grapsa, Frank A Flachskampf, Rafał Dankowski

**Affiliations:** Department of Cardiology, Pomeranian Medical University, Powstańców Wlkp. 72, Szczecin 70-111, Poland; Division of Cardiovascular Imaging, Institute of Heart Diseases, Faculty of Medicine, Wroclaw Medical University, Borowska 213, Wroclaw 50-556, Poland; Department of Cardiac Surgery, Pomeranian Medical University, Powstańców Wlkp. 72, Szczecin 70-111, Poland; Clinical Department of Cardiology, Faculty of Medical Sciences in Zabrze, Medical University of Silesia in Katowice, Specialist Hospital Zabrze, Marii Curie-Skłodowskiej 10, Zabrze 41-800, Poland; Clinical Research Support Center, National Institute of Cardiology, Alpejska 42, Warszawa 04-628, Poland; Department of Cardiology, Medical University of Bialystok, Marii Skłodowskiej-Curie 24a, Białystok 15-276, Poland; Department of Cardiac Surgery, Medical University of Białystok, M. Skłodowskiej-Curie 24a, Białystok 15-276, Poland; Department of Cardiology, Medical University of Lublin, Jaczewskiego 8, 20-090 Lublin, Poland; Department of Cardiology, Centre of Postgraduate Medical Education, Grochowski Hospital, Grenadierów 51/59, 04-073 Warsaw, Poland; Chair and Department of Cardiology, Hypertension and Internal Medicine, Medical University of Warsaw, Mazowiecki Szpital Bródnowski, Kondratowicza 8, 03-258 Warsaw, Poland; 2nd Department of Cardiology, Poznań University of Medical Sciences, 28 Czerwca 1956 r, nr 194, Poznań 61-485, Poland; Department of Valvular Heart Disease, National Institute of Cardiology, Alpejska 42, Warszawa 04-628, Poland; Department of Cardiology, Brigham and Women’s Hospital, Harvard Medical School, 75 Francis St, Boston, MA 02115, USA; Dept. Med. Sciences, Uppsala University, and Clinical Physiology and Cardiology, Uppsala University Hospital, Akademiska Hospital, Uppsala SE-751 85, Sweden; 2nd Department of Cardiology, Poznań University of Medical Sciences, 28 Czerwca 1956 r, nr 194, Poznań 61-485, Poland

## Abstract

**Aims:**

To characterize sex-specific patterns of bicuspid aortic valve (BAV) valvulo-aortopathy using consensus phenotyping and to assess how aortic normalization strategies influence sex-related differences in aortic dimensions.

**Methods and results:**

We analysed 808 adults with BAV (mean age 49.9 ± 17.5 years; 588 men) from the RE-BAV registry. Valve morphology, aortopathy phenotypes, and clinical valvulo-aortopathy categories were classified according to consensus definitions. Typical and uncomplicated forms were additionally analysed together as a non-complex category. Proximal aortic dimensions were defined as the aortic root and ascending aorta.

Valve morphology distribution did not differ by sex. Although the prevalence of aortic dilatation was similar, phenotype distribution differed: women more frequently exhibited the extended phenotype (34.7% vs. 23.3%, *P* = 0.001), whereas the root phenotype was more common in men (15.1% vs. 7.8%, *P* = 0.006). Moderate-to-severe aortic regurgitation was more prevalent in men (50.3% vs. 29.1%, *P* < 0.001). Sex differences in proximal aortic dimensions varied by normalization strategy. For the ascending aorta, indexing to body surface area yielded larger values in women (21.1 ± 4.0 vs. 20.0 ± 4.0 mm/m², *P* = 0.001), whereas diameter/height showed no sex-related difference at this level. Conversely, the cross-sectional area-to-height ratio remained higher in men across proximal segments, most prominently at the sinuses of Valsalva (6.9 ± 2.0 vs. 5.6 ± 1.6 cm²/m, *P* < 0.001). Age was independently associated with larger proximal aortic dimensions across all strategies.

**Conclusion:**

Sex-related differences in BAV involved aortopathy phenotype, aortic regurgitation burden, and proximal aortic dimensions. Interpretation of proximal aortic dimensions depended on the normalization strategy used, indicating that indexing methods should not be treated as interchangeable.

## Graphical Abstract

**Graphical Abstract:**
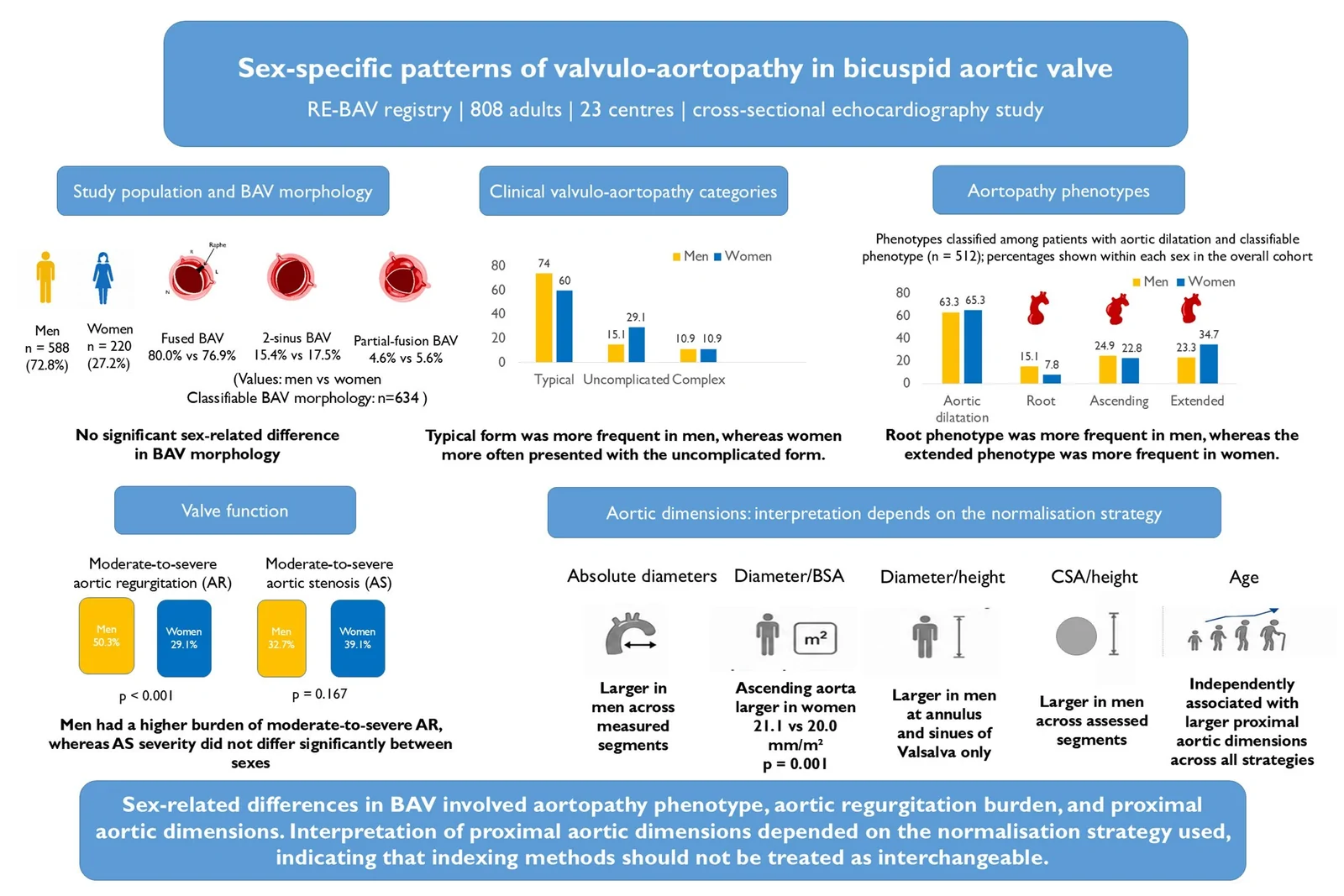

**See the editorial comment for this article ‘Novel insights into sex differences for bicuspid valvulo-aortopathy within the contemporary international consensus’, by H.I. Michelena and A. Evangelista, https://doi.org/10.1093/ehjci/jeag253.**

## Introduction

A bicuspid aortic valve (BAV) is the most common congenital cardiac malformation, affecting approximately 1–2% of the general population, with a higher prevalence in men.^1–4^ Its clinical expression is heterogeneous and reflects the interaction between valve morphology, associated congenital defects, and concomitant aortopathy.^5^ Affected individuals are at increased risk of progressive aortic valve dysfunction, thoracic aortic dilatation, infective endocarditis and aortic dissection.^6,7^

Previous studies have reported sex-related differences in BAV expression, but comparisons across cohorts are limited by heterogeneous classification schemes and differences in aortic measurement and body-size normalization strategies.^2,8–11^ In particular, the interpretation of sex-related differences in aortic dimensions may vary according to whether absolute diameters, diameters indexed to body surface area (BSA), diameters indexed to height, or the cross-sectional area-to-height ratio (CSA/height) are used. These issues have not been fully re-evaluated in a large multicentre cohort using a contemporary international consensus framework.^12^

The RE-BAV multicentre echocardiography registry provides an opportunity to address these gaps through standardized imaging assessment.^13^

Therefore, we aimed to assess sex-specific differences in BAV morphology, aortopathy phenotypes, clinical valvulo-aortopathy categories, and aortic dimensions in the RE-BAV registry and to determine how different aortic normalization strategies influence the interpretation of sex-related differences in aortic dimensions.

## Methods

### Study population

All adult patients (≥18 years) diagnosed with BAV were eligible. Data were prospectively collected between January and December 2023 from 23 academic echocardiography laboratories across Poland. Participating centres were instructed to include consecutive patients undergoing transthoracic echocardiography in whom BAV was identified, both newly diagnosed and those under follow-up in outpatient or inpatient settings. The present analysis was designed as a cross-sectional imaging study; longitudinal follow-up data collected within the registry were not included. The contribution of individual centres ranged from 6 to 164 patients (approximately 0.7–20.3% of the total cohort).

### Echocardiographic assessment

Transthoracic echocardiography was performed in all participants using M-mode, two-dimensional imaging, and Doppler techniques, in accordance with current recommendations.^14^ All examinations were conducted by experienced echocardiographers. Left ventricular (LV) volumes and ejection fraction (LVEF) were assessed using the biplane Simpson method from the apical four- and two-chamber views; if available, three-dimensional echocardiography was also used. Regional wall motion abnormalities were evaluated qualitatively. BAV was confirmed in the parasternal short-axis view. Aortic valve stenosis (AS) or regurgitation (AR) was graded as mild, moderate, or severe using an integrated multiparametric approach.^15,16^

Aortic dimensions were obtained by two-dimensional echocardiography at the level of the annulus (inner-edge to inner-edge, mid-systole), sinuses of Valsalva, sinotubular junction and ascending aorta (leading-edge to leading-edge, end-diastole), according to EACVI recommendations.^17^ The presence of aortic dilatation and aortopathy phenotypes was defined based on absolute aortic diameters, using sex-specific normal reference values: for men, the upper limits of normal were 40 mm at the root and ascending aorta and 34 mm at the arch; for women, the respective cut-offs were 34, 36, and 31 mm.^17^ To address heterogeneity in aortic size normalization, aortic diameters were evaluated using three established body-size–adjustment strategies: BSA indexing, diameter indexing to height, and cross-sectional area (CSA) indexing to height. Diameter/height was calculated as aortic diameter in millimetres divided by height in metres and expressed as mm/m. The CSA/height ratio was calculated as π·(D/2)^2^/height, after converting aortic diameters to centimetres, as used in BAV cohorts.^18,19^

For indexed and multivariable analyses, proximal aortic segments were defined as the aortic root and ascending aorta. These analyses were prespecified to focus on these segments, where BAV-related aortopathy is most pronounced, and data availability was highest.

Based on the location of dilatation, aortopathy was classified as root phenotype (isolated root dilatation), ascending phenotype (tubular dilatation only), or extended phenotype (involving more than one aortic segment, such as root + ascending or ascending + arch). Because the registry did not systematically distinguish specific multi-segment combinations, extended phenotypes were treated as a single category (*Figure 1D*). Transoesophageal echocardiography, computed tomography (CT), or cardiac magnetic resonance (CMR) was performed when clinically indicated.

**Figure 1:**
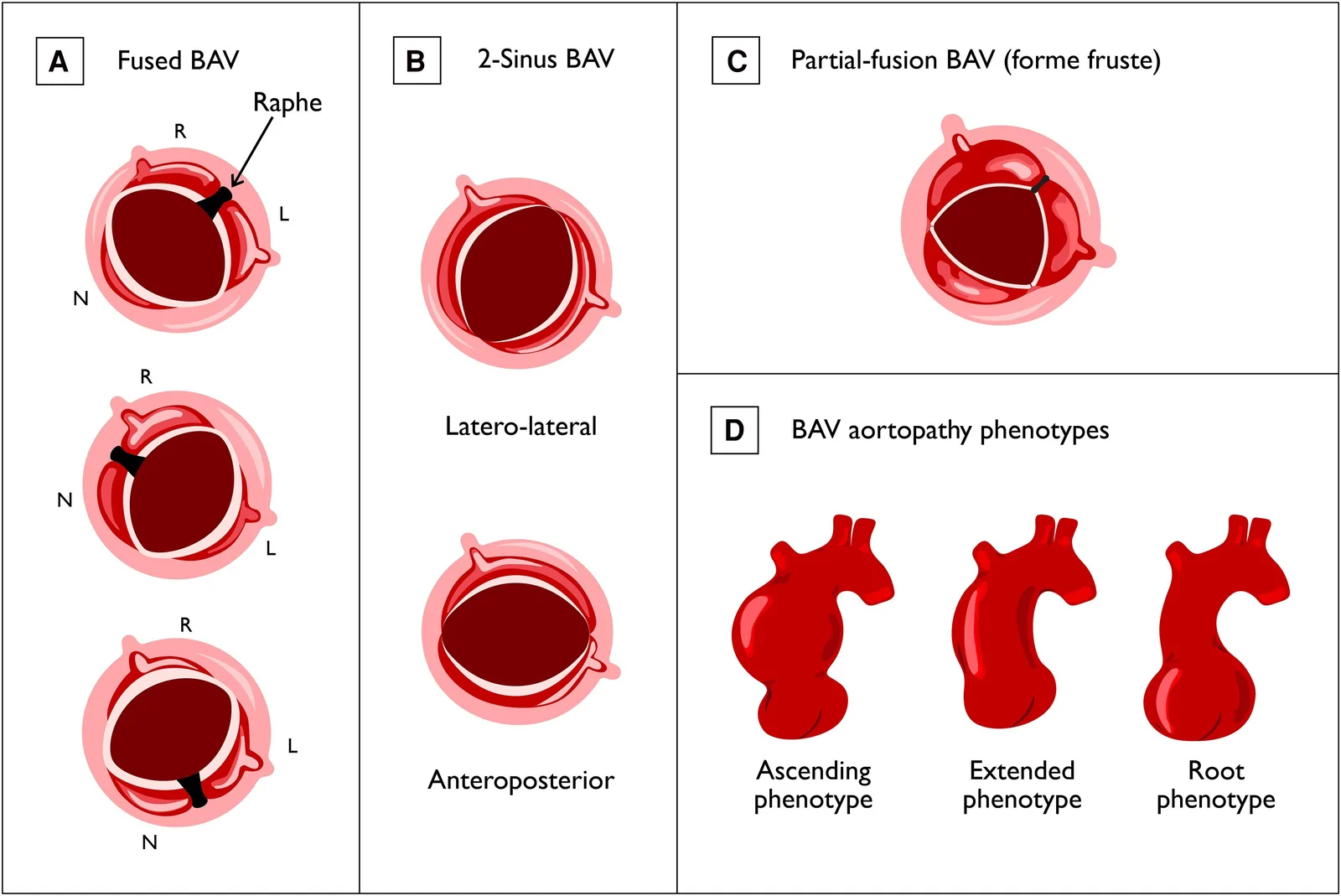
Morphological variants of bicuspid aortic valve and associated aortopathy phenotypes. Representative examples of BAV morphologies and aortic phenotypes: (*A*) Fused BAV, (*B*) 2-sinus BAV, (*C*) Partial-fusion BAV (forme fruste), and (*D*) ascending, extended, and root aortopathy phenotypes. Labels: R—right coronary cusp; L—left coronary cusp; N—non-coronary cusp. BAV morphological classification and aortopathy phenotypes are presented according to the international consensus classification proposed by Michelena et al.^12^. Illustrations were created de novo for the present study.

### Assessment of commissural angle

The commissural angle was measured in diastole using parasternal or transoesophageal short-axis views.^20^ Non-fused commissures were identified, and virtual lines were drawn from each commissure to the valve centre. The angle between these lines was defined as the commissural angle.

### Clinical classification

Patients were categorized into three clinical subgroups according to disease complexity.^12^ Complex valvulo-aortopathy was defined by the presence of associated congenital syndromes (such as Turner syndrome, Loeys–Dietz syndrome, or Shone complex), severe coarctation of the aorta, and/or early and accelerated valvular or aortic disease. Genetic testing was not systematically performed within the registry. Therefore, classification of complex valvulo-aortopathy was based on documented clinical, echocardiographic, and/or previously established syndromic features available in the medical records. Typical valvulo-aortopathy was characterized by progressive aortic valve dysfunction or dilatation in the absence of significant congenital comorbidities. Uncomplicated BAV was defined as an incidental finding, typically with mild or non-progressive valvulo-aortopathy. Because this registry is cross-sectional, the clinical categories were interpreted as phenotypic descriptors rather than longitudinal stages of disease progression. In particular, the uncomplicated group was considered to represent the milder end of the non-complex BAV spectrum, whereas the typical group included patients with clinically recognized valve dysfunction or aortic dilatation in the absence of major associated congenital or syndromic conditions. Accordingly, in addition to the three-category classification, a secondary grouping was performed in which uncomplicated and typical forms were analysed together as non-complex valvulo-aortopathy.

### BAV phenotype classification

BAV morphology was classified into three main types: fused BAV, 2-sinus BAV, and partial-fusion BAV.^20^ In the fused type, two of the three cusps were joined within three separate sinuses, forming two functional cusps. Specific fusion patterns included right–left, right–non-coronary, and left–non-coronary cusp fusion (*Figure 1A*). In the 2-sinus type, two symmetric cusps, each occupying 180° of the annular circumference, formed only two aortic sinuses. This variant was further subclassified into latero-lateral and anteroposterior orientations (*Figure 1B*). In the partial-fusion type (forme fruste), three distinct cusps and sinuses were present, but <50% fusion (mini-raphe) was observed at the base of one commissure, representing a transitional form between tricuspid and bicuspid morphology (*Figure 1C*).

### Statistical analysis

Statistical analyses were performed using SAS version 9.4 (SAS Institute, Cary, NC, USA). The normality of continuous variables was assessed with the Kolmogorov–Smirnov test. Normally distributed data are presented as mean ± standard deviation; non-normally distributed data as median and interquartile range [25th–75th percentiles]. Categorical variables are reported as counts and percentages. Between-group comparisons used the χ^2^ or Fisher’s exact test for categorical variables and Student’s *t*-test (Satterthwaite method) or Wilcoxon rank-sum test for continuous variables, as appropriate. A two-sided *P*-value < 0.05 was considered statistically significant. No missing value imputation techniques were used.

To account for body size, aortic diameters were analysed both as absolute values and after body-size normalization. Indexed analyses were restricted to the aortic root and ascending aorta because of the high proportion of missing measurements in distal segments. Multivariable linear regression models were used to assess the independent associations of age and female sex with proximal aortic dimensions across different indexing strategies. Models included age, sex, height, weight, systolic blood pressure, and hypertension as covariates.

This study was conducted and reported in accordance with the STROBE (Strengthening the Reporting of Observational Studies in Epidemiology) guidelines. It was carried out in accordance with the Declaration of Helsinki and adhered to the recommendations of the International Committee of Medical Journal Editors (ICMJE) and the Committee on Publication Ethics (COPE). All participants provided informed consent to be included in the registry. All data were fully anonymised, and no identifiable patient information was used.

## Results

### Patient demographics and clinical characteristics

The study population consisted of 808 patients, including 588 men (72.8%) and 220 women (27.2%). Baseline characteristics are summarized in *Table 1*. Female patients were slightly older (52.1 ± 17.1 vs. 49.1 ± 17.5 years; *P* = 0.027) and more frequently reported syncope (18/220; 8.5% vs. 26/588; 4.6%; *P* = 0.036). In contrast, men had a higher prevalence of smoking (131/588; 23.8% vs. 25/220; 12.4%; *P* < 0.001) and coronary artery disease (103/588; 18.1% vs. 22/220; 10.4%; *P* = 0.010).

**Table 1:** Baseline clinical characteristics of patients with bicuspid aortic valve, stratified by sex

	All patients (*n* = 808)	Men (*n* = 588)	Women (*n* = 220)	*P* value
Age, y	49.9 ± 17.5	49.1 ± 17.5	52.1 ± 17.1	0.027
Body surface area, m^2^	1.95 ± 0.22	2.01 ± 0.20	1.75 ± 0.18	< 0.001
Body mass index, kg/m^2^	26.8 ± 4.7	27.0 ± 4.6	26.5 ± 5.0	0.265
Heart rate, beats per minute	70 [65–77.0]	70 [64–76]	70 [65–78]	0.882
Systolic blood pressure, mmHg	128.2 ± 16.0	129.3 ± 15.7	125.2 ± 16.3	0.002
Diastolic blood pressure, mmHg	76.9 ± 11.0	77.1 ± 11.1	76.4 ± 10.9	0.473
Smoking, *n* (%)	156 (19.3)	131 (23.8)	25 (12.4)	< 0.001
Coronary artery disease, *n* (%)	125 (15.5)	103 (18.1)	22 (10.4)	0.01
Previous myocardial infarction, *n* (%)	53 (6.6)	42 (7.3)	11 (5.2)	0.288
Stroke, *n* (%)	26 (3.2)	21 (3.7)	5 (2.4)	0.383
Dyslipidaemia, *n* (%)	334 (41.3)	252 (44.8)	82 (39.8)	0.212
Diabetes mellitus, *n* (%)	80 (9.9)	59 (10.5)	21 (10.0)	0.855
Hypertension, *n* (%)	421 (52.1)	313 (55.3)	108 (51.4)	0.336
History of aortic coarctation, *n* (%)	55 (6.8)	38 (6.6)	17 (8.0)	0.518
Previous neoplastic disease, *n* (%)	43 (5.3)	33 (5.8)	10 (4.8)	0.567
Active neoplastic disease, *n* (%)	15 (1.9)	13 (2.3)	2 (1.0)	0.378
Symptoms, *n* (%)				
Syncope, *n* (%)	44 (5.4)	26 (4.6)	18 (8.5)	0.036
Dyspnoea, *n* (%)	431 (53.3)	310 (53.9)	121 (55.8)	0.641
NYHA class				
No symptoms, *n* (%)	355 (44.0)	259 (45.1)	96 (44.2)	0.783
NYHA class—I, *n* (%)	174 (21.5)	128 (22.3)	46 (21.2)	
NYHA class—II, *n* (%)	194 (24.0)	135 (23.5)	59 (27.2)	
NYHA class—III, *n* (%)	58 (7.2)	45 (7.8)	13 (6.0)	
NYHA class—IV, *n* (%)	10 (1.2)	7 (1.2)	3 (1.4)	
Chest pain, *n* (%)	137 (17.0)	95 (16.7)	42 (20.0)	0.292
Marfanoid features, *n* (%)	9 (1.1)	7 (1.2)	2 (0.9)	> 0.99

Values are presented as mean ± standard deviation or number (percentage), as appropriate. Heart rate is shown as median [25th–75th percentile]. *P* values indicate comparisons between men and women.

### Sex-specific prevalence of valvulo-aortopathy forms

The most frequent presentation was the typical form observed in 435/588 men (74.0%) and 132/220 women (60.0%), with a statistically significant difference (*P* < 0.001). The uncomplicated form was more frequent in women (64/220; 29.1%) than in men (89/588; 15.1%; *P* < 0.001). The complex form was the least common, with no significant sex-related difference: 64/588 men (10.9%) vs. 24/220 women (10.9%; *P* = 0.992). Among the 88 patients with complex valvulo-aortopathy, the associated abnormalities included aortic coarctation (*n* = 55, 62.5%), ventricular septal defect (*n* = 17, 19.3%), patent ductus arteriosus (*n* = 12, 13.6%), hypoplastic aortic arch (*n* = 11, 12.5%), atrial septal defect (*n* = 8, 9.1%), transposition of the great arteries and Marfan syndrome (*n* = 3 each, 3.4%), congenital aortic stenosis (*n* = 2, 2.3%), and tetralogy of Fallot and Turner syndrome (*n* = 1 each, 1.1%). Abnormalities were not mutually exclusive. These data are shown in *Figure 2*.

**Figure 2:**
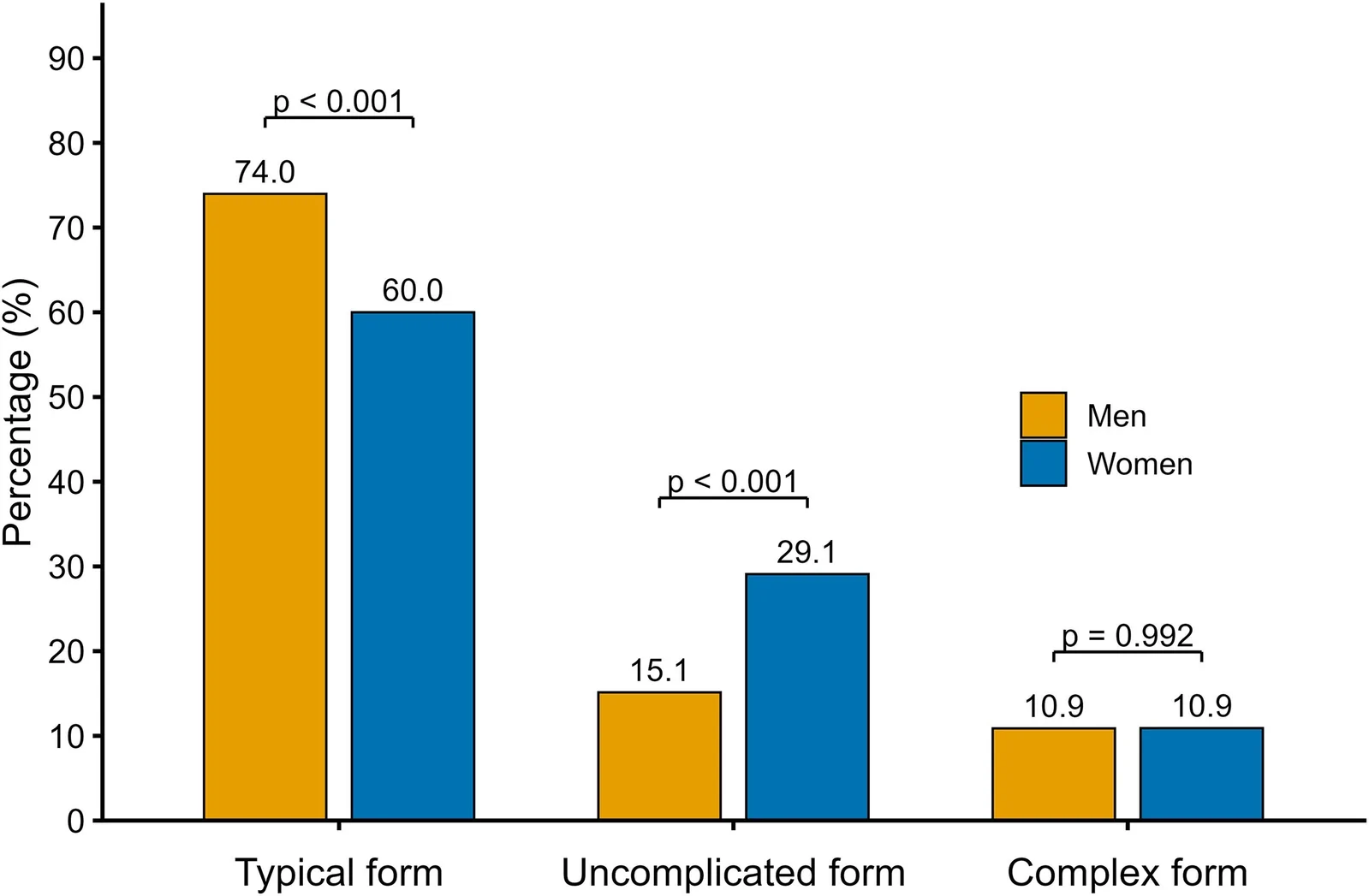
Sex-specific distribution of valvulo-aortopathy forms. Distribution of BAV-associated valvulo-aortopathy forms in men and women. Bars represent percentages within each sex; brackets indicate between-group comparisons.

Because of the cross-sectional study design, typical and uncomplicated forms were combined for a secondary two-category analysis. Non-complex valvulo-aortopathy (typical + uncomplicated) was present in 524/588 (89.1%) of men and 196/220 (89.1%) of women, with no significant sex-related difference (*P* = 0.992).

To further characterize the clinical categories, we compared age and sex distributions, as well as aortic stenosis, aortic regurgitation, and aortopathy phenotype, across the uncomplicated, typical, and complex valvulo-aortopathy groups. The complex group was younger than the uncomplicated and typical groups (34.7 ± 13.6 vs. 49.5 ± 16.0 and 52.4 ± 17.2 years; *P* < 0.001), whereas male sex was most frequent in the typical group (435/567; 76.7%; *P* < 0.001). Moderate-to-severe aortic stenosis and aortic regurgitation were also most frequent in the typical group (262/564; 46.5% and 328/567; 57.8%, respectively; both *P* < 0.001). Detailed data are presented in Supplementary data online, *Table S1*.

### Morphology of BAV

Among 634 individuals with clearly categorized BAV morphology (160 women and 474 men), the fused BAV type was the most prevalent in both sexes, with no significant sex-related difference in its distribution (*P* = 0.701). In patients with fused BAV, right–left cusp fusion predominated, while other fusion patterns were less frequent and similarly distributed between sexes. Likewise, commissural orientation among patients with 2-sinus BAV did not differ significantly between women and men. Detailed distributions are provided in Supplementary data online, *Figure S1*.

### BAV aortopathy phenotypes

Aortic dilatation was observed with comparable frequency in women and men (143 women (65.3%) vs. 369 men (63.3%); *P* = 0.599). Among patients with aortic dilatation and classifiable aortopathy phenotype (*n* = 512), the distribution of aortopathy phenotypes differed by sex. The root phenotype was more frequent in men than in women (88 men (15.1%) vs. 17 women (7.8%); *P* = 0.006), whereas the extended phenotype was more frequent in women than in men (76 women (34.7%) vs. 136 men (23.3%); *P* = 0.001). The ascending phenotype was similarly distributed between sexes (145 men (24.9%) vs. 50 women (22.8%); *P* = 0.548). These data are shown in *Figure 3*.

**Figure 3:**
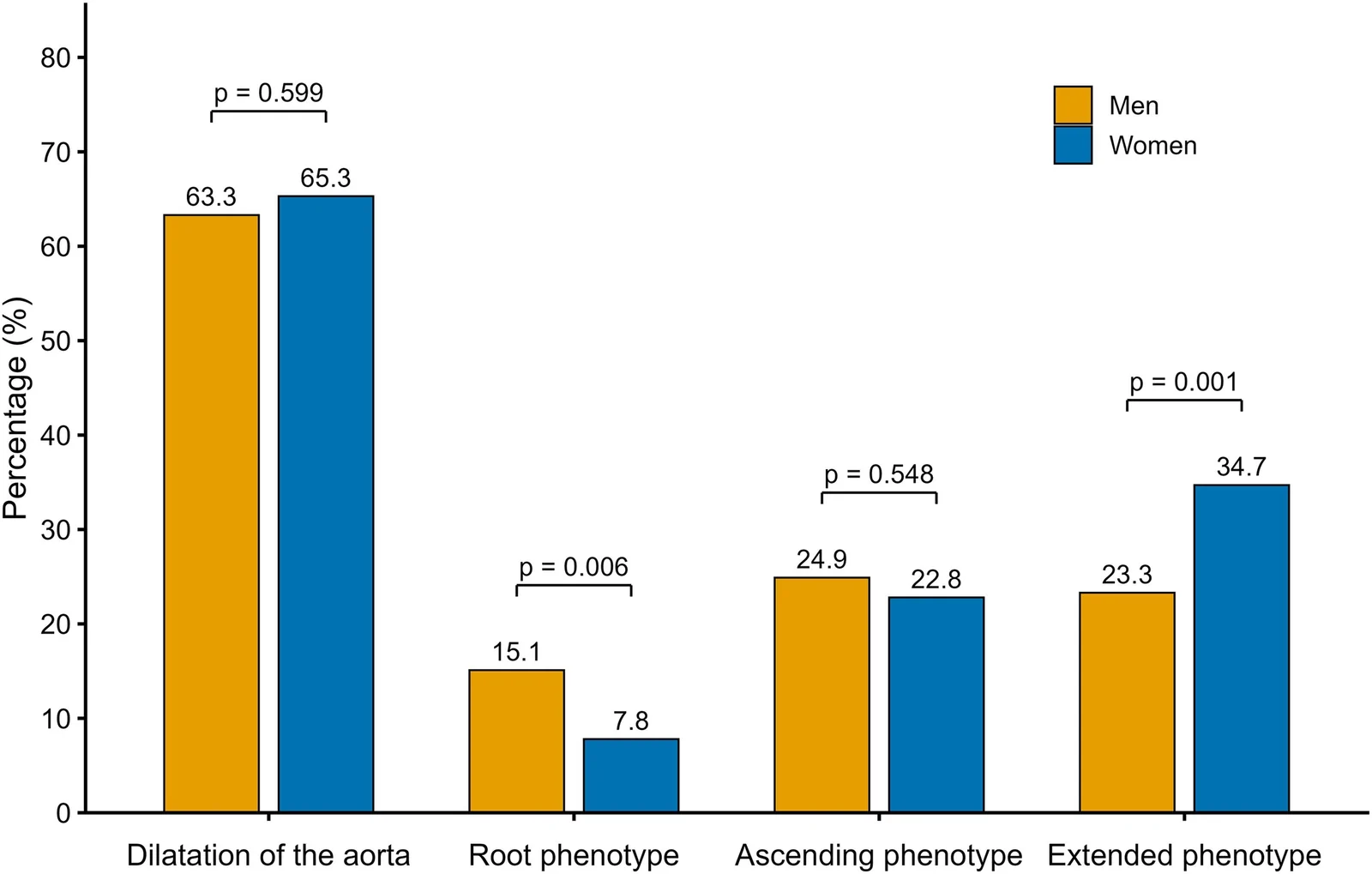
Sex-specific distribution of aortopathy phenotypes. Distribution of overall aortic dilatation and root, ascending, and extended aortopathy phenotypes, stratified by sex. Aortopathy phenotypes were classified among patients with aortic dilatation and a classifiable phenotype. Bars represent percentages within each sex; brackets indicate between-group comparisons. Percentages are reported within each sex in the overall cohort; phenotype categories were assigned to patients with aortic dilatation and a classifiable phenotype.

### Dimensions of the aorta

Men had larger absolute aortic diameters across all measured segments, including the sinuses of Valsalva (39.1 ± 5.6 vs. 33.9 ± 5.3 mm; *P* < 0.001) and ascending aorta (39.7 ± 7.5 vs. 36.9 ± 7.3 mm; *P* < 0.001). After indexing to BSA, sex-related differences were no longer observed at the annulus, sinuses of Valsalva, or sinotubular junction, whereas the ascending aorta indexed to BSA was larger in women than in men (21.1 ± 4.0 vs. 20.0 ± 4.0 mm/m^2^; *P* = 0.001). When aortic diameters were indexed to height, sex-related differences persisted at the sinuses of Valsalva (22.1 ± 3.2 vs. 20.8 ± 3.0 mm/m; *P* < 0.001), but were no longer observed at the sinotubular junction or ascending aorta. In contrast, CSA/height remained larger in men at the sinuses of Valsalva (6.9 ± 2.0 vs. 5.6 ± 1.6 cm^2^/m; *P* < 0.001) and ascending aorta (7.3 ± 2.8 vs. 6.8 ± 2.6 cm^2^/m; *P* = 0.029). Full absolute and indexed values are presented in *Table 2*.

**Table 2:** Absolute and indexed aortic dimensions in patients with bicuspid aortic valve, stratified by sex

	All patients (*n* = 808)	Men (*n* = 588)	Women (*n* = 220)	*P* value
Absolute dimensions of the aorta (mm)				
Annulus	25.1 ± 3.7	25.9 ± 3.6	23.0 ± 3.2	< 0.001
Sinuses of Valsalva	37.7 ± 5.9	39.1 ± 5.6	33.9 ± 5.3	< 0.001
Sinotubular junction	32.3 ± 5.8	33.1 ± 5.7	29.7 ± 5.4	< 0.001
Ascending aorta	39.0 ± 7.5	39.7 ± 7.5	36.9 ± 7.3	< 0.001
Aortic arch	28.8 ± 5.7	29.3 ± 5.8	27.3 ± 5.1	< 0.001
Descending aorta	22.9 ± 4.2	23.3 ± 4.1	21.5 ± 4.2	0.002
Aortic dimensions indexed to BSA (diameter/BSA; mm/m^2^)				
Annulus	13.0 ± 2.0	13.0 ± 2.0	13.1 ± 1.9	0.461
Sinuses of Valsalva	19.5 ± 2.9	19.5 ± 3.0	19.4 ± 2.8	0.536
Sinotubular junction	16.6 ± 3.0	16.5 ± 3.0	16.8 ± 2.9	0.295
Ascending aorta	20.2 ± 4.0	20.0 ± 4.0	21.1 ± 4.0	0.001
Aortic dimensions indexed to height (diameter/height; mm/m)				
Annulus	14.5 ± 2.0	14.7 ± 2.1	14.0 ± 1.8	0.002
Sinuses of Valsalva	21.8 ± 3.2	22.1 ± 3.2	20.8 ± 3.0	< 0.001
Sinotubular junction	18.6 ± 3.2	18.7 ± 3.3	18.1 ± 3.1	0.100
Ascending aorta	22.6 ± 4.4	22.6 ± 4.4	22.6 ± 4.3	0.926
Cross-sectional area of the aorta indexed to height (CSA/height; cm^2^/m)				
Annulus	2.9 ± 0.9	3.0 ± 0.9	2.6 ± 0.7	< 0.001
Sinuses of Valsalva	6.6 ± 2.0	6.9 ± 2.0	5.6 ± 1.6	< 0.001
Sinotubular junction	4.9 ± 1.8	5.0 ± 1.8	4.4 ± 1.5	0.001
Ascending aorta	7.2 ± 2.8	7.3 ± 2.8	6.8 ± 2.6	0.029

Data are presented as mean ± standard deviation. Aortic diameters were measured by two-dimensional echocardiography at standard anatomical landmarks. *P* values indicate comparisons between men and women. All measurements were obtained using transthoracic echocardiography. Abbreviations: BSA, body surface area; CSA, cross-sectional area.

In multivariable linear regression models, age was independently associated with larger sinuses of Valsalva and ascending aorta dimensions across all indexing strategies. For absolute diameters, the age coefficient was β = 0.06 mm/year for the sinuses of Valsalva and β = 0.13 mm/year for the ascending aorta (both *P* < 0.001), with consistent associations observed after BSA, diameter/height, and CSA/height indexing. Female sex remained independently associated with smaller sinuses of Valsalva dimensions across all indexing strategies (absolute diameter: β = −2.74 mm; *P* < 0.001), whereas associations between female sex and ascending aortic dimensions were weak and non-significant after adjustment. These findings are presented in *Table 3*.

**Table 3:** Multivariable associations of age and female sex with proximal aortic dimensions according to indexing strategy

Aortic segment	Predictor	Absolute dimension β (SE), *P* value	Diameter/BSA β (SE), *P* value	Diameter/height β (SE), *P* value	CSA/height β (SE), *P* value
Sinuses of Valsalva	Age, per year	0.06 (0.02), *P* < 0.001	0.03 (0.01), *P* < 0.001	0.035 (0.009), *P* < 0.001	0.02 (0.01), *P* < 0.001
	Female sex	−2.74 (0.64), *P* < 0.001	−1.42 (0.33), *P* < 0.001	−1.64 (0.37), *P* < 0.001	−0.98 (0.23), *P* < 0.001
Ascending aorta	Age, per year	0.13 (0.02), *P* < 0.001	0.07 (0.01), *P* < 0.001	0.073 (0.011), *P* < 0.001	0.04 (0.01), *P* < 0.001
	Female sex	−1.24 (0.85), *P* = 0.144	−0.61 (0.44), *P* = 0.158	−0.74 (0.49), *P* = 0.129	−0.47 (0.31), *P* = 0.135

Each model included age, sex, height, weight, systolic blood pressure, and hypertension as covariates. β coefficients are presented with standard errors and represent the independent association of age or female sex with proximal aortic dimensions. Age effects are expressed per 1-year increase. Female sex was coded relative to male sex as the reference category. BSA, body surface area; CSA, cross-sectional area; SE, standard error.

### Aortic valve characteristics and function

Aortic stenosis data were available in 804 patients (584 men, 220 women). The prevalence of AS did not differ significantly between sexes. Moderate-to-severe AS was present in 86/220 women (39.1%) and 191/584 men (32.7%), while 134/220 women (60.9%) and 393/584 men (67.3%) had either no stenosis or mild stenosis (*P* = 0.167). The detailed distribution of AS severity is shown in Supplementary data online, *Figure S2*.

Women exhibited slightly higher aortic valve peak velocities (2.47 [1.84–3.70] vs. 2.20 [1.64–3.50] m/s; *P* = 0.020) and transvalvular gradients (maximum gradient: 23.0 [14.0–55.0] vs. 19.8 [11.0–49.0] mmHg; *P* = 0.031) than men. Absolute aortic valve area was smaller in women (1.10 [0.73–1.65] vs. 1.30 [0.90–2.20] cm^2^; *P* = 0.002), but this difference was not statistically significant after indexing to BSA (0.60 [0.43–0.89] vs. 0.68 [0.45–1.08] cm^2^/m^2^; *P* = 0.135). Valve area was calculated only in patients with moderate or severe stenosis (*Table 4*).

**Table 4:** Aortic valve haemodynamics and commissural angle in patients with bicuspid aortic valve, stratified by sex

	All patients (*n* = 804)	Men (*n* = 584)	Women (*n* = 220)	*P* value
Peak velocity, m/s	2.30 [1.70–3.50]	2.20 [1.64–3.50]	2.47 [1.84–3.70]	0.020
Maximum gradient, mmHg	21.0 [11.5–50]	19.8 [11.0–49.0]	23.0 [14.0–55.0]	0.031
Mean gradient, mmHg	13.0 [6.4–34.0]	12.0 [6.0–33.0]	15.0 [8.0–35.0]	0.056
Aortic valve area, cm^2^	1.23 [0.90–2.0]	1.3 [0.90–2.20]	1.1 [0.73–1.65]	0.002
Indexed aortic valve area, cm^2^/m^2^	0.64 [0.44–1.01]	0.68 [0.45–1.08]	0.60 [0.43–0.89]	0.135
Commissural angle, degrees	160.2 ± 17.9	159.1 ± 18.5	163.9 ± 15.1	0.011

Values are presented as median [25th–75th percentile] or mean ± standard deviation, as appropriate. Haemodynamic parameters were assessed by Doppler echocardiography.

Peak velocity and gradients are reported for all patients with available data (*n* = 804); aortic valve area and indexed area were calculated only in patients with moderate or severe aortic stenosis (AS grades 2–3). *P* values indicate comparisons between men and women.

Aortic regurgitation data were available in 805 patients (585 men, 220 women). In contrast, aortic regurgitation showed a clear sex-related pattern. Moderate-to-severe regurgitation was more common in men (294/585; 50.3%) than in women (64/220; 29.1%; *P* < 0.001), whereas absent or mild regurgitation was more frequent in women (156/220; 70.9%) than in men (291/585; 49.7%; *P* < 0.001). The detailed distribution of AR severity is shown in *Figure 4*.

**Figure 4:**
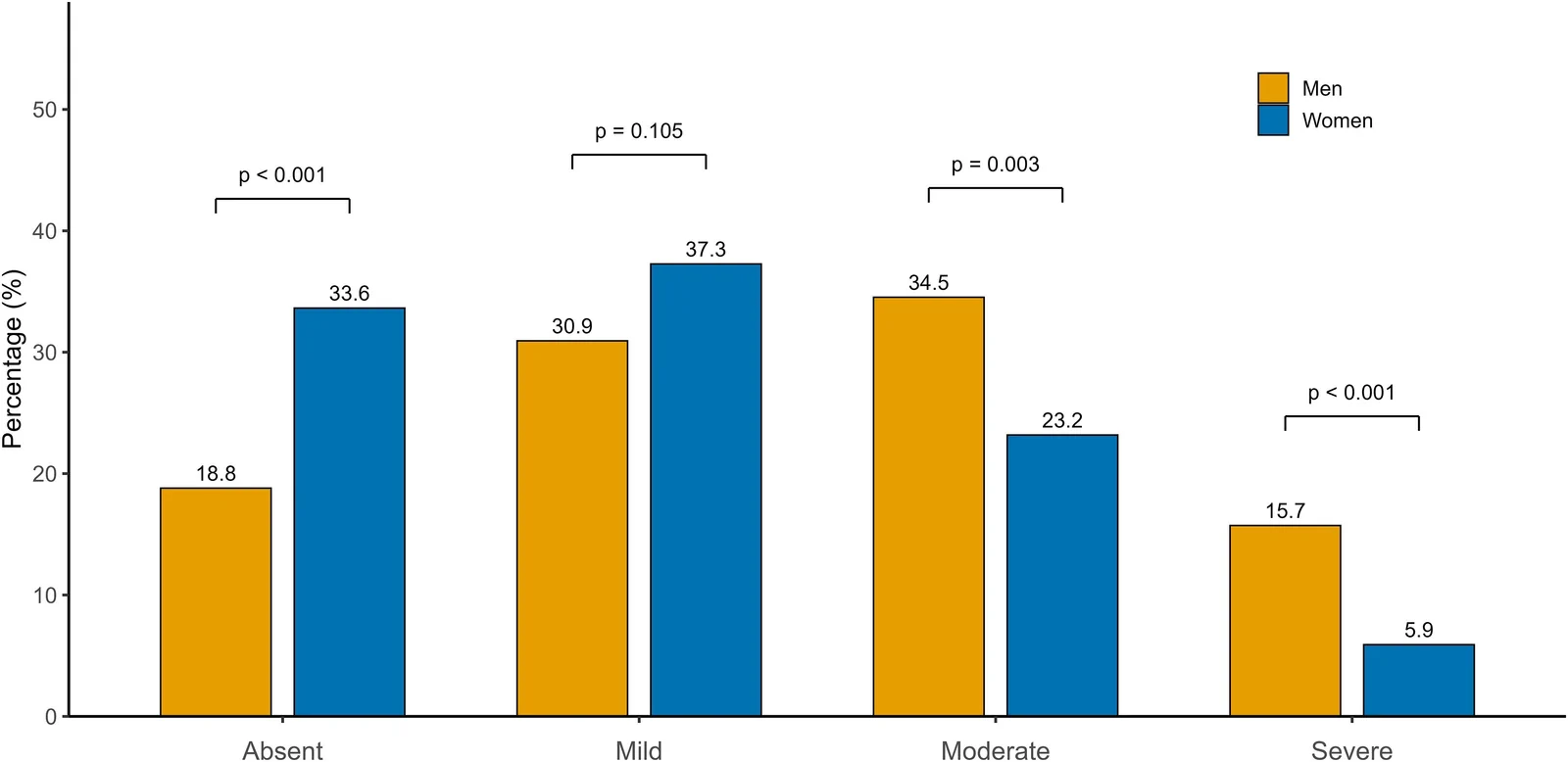
Severity of aortic regurgitation by sex. Distribution of aortic regurgitation severity in men and women. Bars represent percentages within each sex; brackets indicate between-group comparisons.

The commissural angle was significantly larger in women (163.9 ± 15.1°) than in men (159.1 ± 18.5°; *P* = 0.011).

### Echocardiographic characteristics of cardiac chambers and ventricular function

Men exhibited significantly larger left ventricular end-diastolic and end-systolic volumes than women (143.5 vs. 85.0 mL and 67.0 vs. 37.0 mL, respectively; both *P* < 0.001), with similar differences after indexing to BSA. In contrast, women demonstrated higher LVEF (60.1% vs. 56.7%; *P* < 0.001) and greater (i.e. more negative) global longitudinal strain (GLS) (−18.2% vs. −16.4%; *P* < 0.001). Regional wall motion abnormalities were observed more frequently in men than in women (13.2% vs. 5.5%; *P* = 0.002). Detailed echocardiographic measurements are presented in Supplementary data online, *Table S2*.

## Discussion

In this large, multicentre echocardiographic registry analysed according to the international consensus framework, several sex-related patterns of BAV expression were observed. First, the distribution of BAV morphological types did not differ significantly between women and men. Second, aortopathy phenotype differed by sex, with root phenotype being more frequent in men and extended phenotype being more frequent in women. Third, the interpretation of proximal aortic dimensions depended on the normalization strategy, with BSA indexing, diameter/height, and CSA/height yielding different sex-related patterns. Fourth, age was independently associated with larger proximal aortic dimensions across all indexing strategies. Fifth, men had a higher prevalence of moderate-to-severe aortic regurgitation, whereas aortic stenosis severity was comparable between sexes. Finally, men showed more pronounced LV remodelling and less favourable systolic function, interpreted primarily in the context of aortic valve dysfunction.

### BAV aortopathy, phenotypes and dilatation of the aorta

When interpreted within a consensus-based phenotypic framework,^12^ sex-related differences in BAV-associated aortopathy were observed mainly in the anatomical pattern of aortic involvement rather than in the overall prevalence of aortic dilatation. Men more frequently exhibited the root phenotype, whereas women more often presented with the extended phenotype. This pattern is broadly consistent with previous BAV cohorts linking male sex with aortic root involvement and AR and female sex with stenotic valve disease or ascending aortic involvement.^2,21,22^

A central finding of the present analysis is that sex-related differences in aortic dimensions depended on the normalization strategy applied. Absolute diameters emphasized larger aortic dimensions in men, whereas BSA indexing attenuated most proximal sex-related differences and shifted the relative pattern for the ascending aorta towards women. This likely reflects the fact that BSA incorporates both height and weight and may therefore overcorrect aortic size in larger-bodied individuals, particularly men. Diameter/height, which is less influenced by body weight, preserved some but not all sex-related differences, whereas CSA/height yielded a different pattern because cross-sectional area increases with the square of diameter. These findings indicate that diameter/height and CSA/height should not be treated as interchangeable and that interpretation of sex-related aortic differences in BAV should explicitly account for the normalization strategy used.^11,19,23,24^

These observations are consistent with normative echocardiographic data showing that age, sex, and body size influence proximal aortic dimensions, and that BSA- and height-based normalization may yield sex-specific interpretations. In this cohort, age remained independently associated with larger sinuses of Valsalva and ascending aortic dimensions across all normalization strategies. These findings support the inclusion of age in models evaluating BAV-associated aortopathy, but do not establish one indexing method as prognostically superior.^22–24^

### Clinical classification and disease complexity

Application of the international consensus clinical categories showed sex-related differences in the distribution of valvulo-aortopathy forms. The typical form was more frequent in men, whereas women were more often represented in the uncomplicated category. The complex form was uncommon and did not differ significantly between sexes.

These findings should be interpreted cautiously because the registry was cross-sectional. The uncomplicated, typical, and complex categories describe phenotypes at the time of inclusion rather than longitudinal stages of BAV progression. Therefore, differences between typical and uncomplicated forms may partly reflect referral pathways, timing of diagnosis, and clinical recognition, rather than intrinsic sex-specific disease progression.

To address this limitation, uncomplicated and typical forms were additionally analysed together as non-complex valvulo-aortopathy; in this secondary grouping, non-complex vs. complex disease did not differ between women and men. Supplementary data online, *Table S1* further characterizes valve dysfunction and aortopathy phenotype across these clinical categories.

### Valve morphology and valve function

The distribution of BAV morphological types did not differ significantly between women and men. The fused BAV type predominated in both sexes, with similar distributions of fusion patterns and commissural orientations. It suggests that the observed sex-related differences in aortopathy phenotype, valve dysfunction, and LV remodelling are unlikely to be explained by major differences in valve morphology alone.^2,21^

Aortic stenosis severity was comparable between sexes using integrated echocardiographic criteria. Although women had slightly higher velocities and gradients and smaller absolute valve areas, indexed valve area and categorical stenosis severity did not differ significantly. These findings should be interpreted as cross-sectional haemodynamic differences rather than evidence of sex-specific stenosis progression.

In contrast, aortic regurgitation showed a clear sex-related pattern, with moderate-to-severe AR being substantially more frequent in men. This observation is consistent with previous BAV cohorts and may partly reflect the greater frequency of the root phenotype and larger root dimensions in men.^2,21,22,25^ However, because longitudinal data were not analysed, these associations should not be interpreted as evidence of divergent valve-disease trajectories. Women also had a slightly larger commissural angle, but the clinical relevance of this small difference remains uncertain.

### Left ventricular function and remodelling

Men demonstrated larger LV volumes, lower LVEF, less favourable GLS, and more frequent regional wall motion abnormalities, indicating more pronounced LV remodelling and subtly less favourable systolic function. These differences should be interpreted primarily in relation to the higher prevalence of moderate-to-severe AR in men, which may contribute to chronic volume overload and LV dilatation. Because this was a cross-sectional analysis, causal links between valve dysfunction, aortic phenotype, and ventricular remodelling cannot be established. The absence of major sex-related differences in NYHA class indicates that symptom class may not fully reflect structural and functional differences at inclusion.^2,21^

### Limitations

Several limitations of this study should be acknowledged. First, this registry-based cohort may not represent the full spectrum of BAV in the general population, particularly asymptomatic or incidentally detected cases. Women accounted for approximately one-quarter of the cohort, reflecting the known male predominance of BAV^4^ but potentially limiting selected sex-specific comparisons.

Second, the cross-sectional design precludes assessment of disease progression, temporal changes in aortic dimensions, or clinical outcomes. Accordingly, the observed sex-related differences should be interpreted as phenotypic and anatomical patterns rather than prognostic trajectories. The uncomplicated, typical, and complex valvulo-aortopathy categories should likewise be regarded as cross-sectional clinical descriptors rather than longitudinal stages.

Third, transthoracic echocardiography was the primary imaging modality. Although examinations were performed in experienced centres, echocardiography has limitations in visualizing distal aortic segments, and CT or CMR was performed only when clinically indicated.

Fourth, genetic testing was not systematically performed. Classification of complex valvulo-aortopathy was therefore based on documented clinical, echocardiographic, and previously established syndromic features, and some genetically mediated or syndromic forms may have been under-recognized.

Finally, although several aortic size normalization strategies were compared, outcome data were not analysed; therefore, this study cannot determine which method provides the strongest prognostic information. Missing data affected selected variables, and no imputation was performed. Despite these limitations, the large sample size, multicentre design, and application of the international consensus framework strengthen the interpretation of the observed associations.

## Conclusions

In this large, multicentre echocardiographic registry analysed according to the international consensus framework, sex-related differences in BAV involved aortic phenotype, valve dysfunction, proximal aortic dimensions, and LV remodelling, but not the distribution of BAV morphological types. Men more frequently exhibited the root phenotype, moderate-to-severe AR, and more pronounced LV remodelling, whereas women more often presented with extended aortopathy. The interpretation of proximal aortic dimensions depended on the normalization strategy applied, indicating that aortic indexing methods should not be treated as interchangeable. Longitudinal studies are needed to determine how these patterns relate to aortic growth, valve-disease progression, clinical outcomes, and intervention thresholds.

## Supplementary Material

jeag233_Supplementary_Data

## Data Availability

The de-identified data underlying the findings of this study are available from the first author upon reasonable request, subject to applicable ethical, legal, and institutional restrictions.

## Supplementary data

Supplementary material includes two tables and two figures that provide additional clinical and echocardiographic characteristics of patients with a bicuspid aortic valve. Supplementary data online, *Table S1* presents clinical and echocardiographic characteristics by valvulo-aortopathy category. Supplementary data online, *Table S2* presents detailed measurements of cardiac volumes and ventricular function. Supplementary data online, *Figures S1*–*S2* illustrate sex-specific distributions of BAV morphology and cusp fusion patterns, and the severity of aortic stenosis.
