# Supplementary material for: Sex-specific patterns of valvulo-aortopathy in bicuspid aortic valve according to the international consensus framework: insights from the RE-BAV registry
Source: Eur Heart J Cardiovasc Imaging. 2026 Aug 31;27(11):1926–36. doi: 10.1093/ehjci/jeag233 (PMC13721766; doi:10.1093/ehjci/jeag233)
Supplement: Supplementary file 1 [file jeag233_supplementary_data.zip › FINAL BAV_supplementary_material_production_26aug2026.docx]

Supplementary Table S1. Clinical and Echocardiographic Characteristics According to Valvulo-Aortopathy Category.

|  | Uncomplicated | Typical | Complex | p value | Pairwise comparisons |
| --- | --- | --- | --- | --- | --- |
| Number of patients | 153 | 567 | 88 |  |  |
| Age, years | 49.5 ± 16.0 | 52.4 ± 17.2 | 34.7 ± 13.6 | < 0.001 | Uncomplicated vs complex: p < 0.001; typical vs complex: p < 0.001 |
| Male sex | 89 (58.2%) | 435 (76.7%) | 64 (72.7%) | < 0.001 | Uncomplicated vs typical: p < 0.001; uncomplicated vs complex: p = 0.024 |
| Aortic stenosis severity, n (%)  (available in 804 patients: uncomplicated n = 153, typical n = 564, complex n = 87) | | | | < 0.001 | All pairwise comparisons p < 0.001 |
| None | 121 (79.1%) | 235 (41.7%) | 62 (71.3%) |  |  |
| Mild | 32 (20.9%) | 67 (11.9%) | 10 (11.5%) |  |  |
| Moderate | 0 (0%) | 113 (20.0%) | 7 (8.1%) |  |  |
| Severe | 0 (0%) | 149 (26.4%) | 8 (9.2%) |  |  |
| Aortic regurgitation severity, n (%)  (available in 805 patients: uncomplicated n = 152, typical n = 567, complex n = 86) | | | | < 0.001 | All pairwise comparisons p < 0.001 |
| None | 74 (48.7%) | 94 (16.6%) | 16 (18.6%) |  |  |
| Mild | 75 (49.3%) | 145 (25.6%) | 43 (50.0%) |  |  |
| Moderate | 2 (1.3%) | 229 (40.4%) | 22 (25.6%) |  |  |
| Severe | 1 (0.7%) | 99 (17.5%) | 5 (5.8%) |  |  |
| Aortopathy phenotype among patients with aortic dilatation and classifiable phenotype, n (%)  (available in 512 patients: uncomplicated n = 70, typical n = 402, complex n = 40) | | | | < 0.001 | Uncomplicated vs typical: p = 0.001; typical vs complex: p = 0.023 |
| Ascending phenotype | 33 (47.1%) | 149 (37.1%) | 13 (32.5%) |  |  |
| Root phenotype | 21 (30.0%) | 70 (17.4%) | 14 (35.0%) |  |  |
| Extended phenotype | 16 (22.9%) | 183 (45.5%) | 13 (32.5%) |  |  |

Data are presented as mean ± standard deviation or n (%). Pairwise post hoc comparisons are shown where available.

Supplementary Table S2.

Echocardiographic Measurements of Cardiac Volumes and Ventricular Function in Patients with Bicuspid Aortic Valve, Stratified by Sex.

|  | All patients, n = 808 | Women, n = 220 | Men, n = 588 | P value |
| --- | --- | --- | --- | --- |
| Left ventricular end-systolic volume, ml | 60 [37–91] | 37 [26–58] | 67 [43–100] | < 0.001 |
| Indexed left ventricular end-systolic volume, ml/m^2^ | 30.1 [19.0–45.4] | 20.6 [14.9–32.6] | 32.5 [22.0–50.3] | < 0.001 |
| Left ventricular end-diastolic volume, ml | 127.0 [89.0 –180.0] | 85 [63.5–116.5] | 143.5 [106.2–193] | < 0.001 |
| Indexed left ventricular end-diastolic volume, ml/m^2^ | 65.4 [46.2–89.8] | 47 [35.9–65.7] | 70.1 [52.2 –92.8] | < 0.001 |
| Indexed left atrial volume, ml/m^2^ | 19.4 [11.8–31.9] | 21.3 [11.3–32.0] | 19.3 [12.3–31.9] | 0.855 |
| LVEF, % | 57.7 ± 10.3 | 60.1 ± 8.5 | 56.7 ± 10.9 | < 0.001 |
| Regional wall motion abnormalities, n (%) | 87 (10.8) | 12 (5.5) | 75 (13.2) | 0.002 |
| LV global longitudinal strain, % | −16.8 ± 3.7 | −18.2 ± 3.3 | −16.4 ± 3.7 | < 0.001 |

Values are presented as median [25th–75th percentile] or mean ± standard deviation, as appropriate; categorical variables as n (%). Indexed values are normalised to body surface area. P values indicate comparisons between men and women. Abbreviations: LV = left ventricle; LVEF = left ventricular ejection fraction.

Supplementary Figures.

Supplementary Figure S1.

Sex-specific distribution of BAV morphology and cusp fusion patterns.

Panel A shows overall BAV morphology among patients with defined valve morphology (men: n = 474; women: n = 160). Panel B presents cusp fusion patterns among patients with fused BAV (men: n = 334; women: n = 109). Panel C illustrates commissural orientation among patients with 2-sinus BAV (men: n = 67; women: n = 23).

Bars represent percentages within each sex. No statistically significant sex-related differences were observed.

Supplementary Figure S2.

Severity of aortic stenosis by sex.

Distribution of aortic stenosis severity (absent, mild, moderate, severe) in women and men. Bars represent percentages within each sex. No statistically significant sex-related differences were observed.
